# Interpreting vision and language generative models with semantic visual priors

**DOI:** 10.3389/frai.2023.1220476

**Published:** 2023-09-25

**Authors:** Michele Cafagna, Lina M. Rojas-Barahona, Kees van Deemter, Albert Gatt

**Affiliations:** ^1^Institute of Linguistics and Language Technology, University of Malta, Msida, Malta; ^2^Orange Innovation, Lannion, France; ^3^Department of Information and Computing Sciences, Utrecht University, Utrecht, Netherlands

**Keywords:** vision and language, multimodality, explainability, image captioning, visual question answering, natural language generation

## Abstract

When applied to Image-to-text models, explainability methods have two challenges. First, they often provide token-by-token explanations namely, they compute a visual explanation for each token of the generated sequence. This makes explanations expensive to compute and unable to comprehensively explain the model's output. Second, for models with visual inputs, explainability methods such as SHAP typically consider superpixels as features. Since superpixels do not correspond to semantically meaningful regions of an image, this makes explanations harder to interpret. We develop a framework based on SHAP, that allows for generating comprehensive, meaningful explanations leveraging the meaning representation of the output sequence as a whole. Moreover, by exploiting semantic priors in the visual backbone, we extract an arbitrary number of features that allows the efficient computation of Shapley values on large-scale models, generating at the same time highly meaningful visual explanations. We demonstrate that our method generates semantically more expressive explanations than traditional methods at a lower compute cost and that it can be generalized to a large family of vision-language models.

## 1. Introduction

Multimodal learning research has witnessed a surge of effort leading to substantial improvements, in algorithms involving the integration of vision and language (V&L ), for tasks such as image captioning (Lin et al., 2014; Hossain et al., 2019; Sharma et al., 2020) and visual question answering (Antol et al., 2015; Zhu et al., 2016; Srivastava et al., 2021). The need has arisen to create more challenging tasks and benchmarks requiring higher fine-grained linguistic capabilities (Parcalabescu et al., 2022; Thrush et al., 2022; Li et al., 2023) and semantic and temporal understanding (Yu et al., 2016; Park et al., 2020).

In this context, the role of interpretability methods has become central to assessing the models' grounding capabilities. However, such tools are often designed for specific classes of tasks or models. To overcome this limitation, model-agnostic interpretability methods, such as SHAP-based methods (Lundberg and Lee, 2017), are often preferred over others, since they rely on a solid theory and benefit from desirable properties not available in other methods.

When such methods are applied to V&L generative tasks, like image-captioning, the goal is to explain the textual output with reference to the visual input. However, the text generation process happens token-by-token, and as a result, most of the interpretability methods applied in this context tend to produce local token-by-token explanations. Moreover, for most applications, current methods build the explanation on top of arbitrary regions of the visual input, usually considering superpixels (regions of adjacent pixels of a fixed size) as the features against which to interpret the outputs (Parcalabescu and Frank, 2022).

Token-by-token explanations are hard to interpret as they are token-specific, and they are costly to compute since the number of model evaluations grows exponentially with the number of features used in each explanation. To mitigate these issues, approximation techniques, like sampling, and input feature reduction are usually applied. However, this produces inaccurate explanations which lack detail and are hard to interpret. Furthermore, the reliance on superpixels as input features makes interpretation harder since superpixels do not necessarily correspond to semantically meaningful regions of an image.

In this work, we address these issues by proposing:

1. A modular framework to create a new family of tools to generate explanations in V&L generative settings;
2. A method to generate sentence-based explanations for vision-to-text generative tasks, as opposed to token-by-token explanations, showing that such explanations can efficiently be generated with SHAP by exploiting semantic knowledge from the two modalities;
3. A method to reduce the number of visual input features by exploiting the semantics embedded in the models' visual backbone. We extend this method to a number of different architectures. We further propose an alternative approach to extract semantically meaningful features from images in case a model architecture does not support our specific method;
4. A human evaluation designed to assess key user-centric properties of our explanations.

## 2. Related work

In this section, we discuss related work on interpretable machine learning and explainable AI (XAI) in Vision and Language models. We also detail some of the essential properties of the XAI framework (SHAP) on which we base our own work.

### 2.1. Interpretable machine learning

Interpretable machine learning is a multidisciplinary field encompassing efforts from computer science, human-computer interaction, and social science, aiming to design user-oriented and human-friendly explanations for machine learning models. It plays an important role in the field for a series of reasons: it increases trust, confidence, and acceptance of machine learning models by users, and enables verification, validation, and debugging of machine learning models. Techniques for deep neural networks (DNN) can be grouped into two main categories: *white-box* methods which exploit the knowledge of the internal structure of the model to generate the explanation and *black-box* methods, also called model-agnostic, which operate only on the inputs and the outputs (Loyola-Gonzalez, 2019).

*White-box methods* There exist two types of white-box methods: attention-based and gradient-based methods. *Attention-based* methods (e.g., Ahmed et al., 2021; Zheng et al., 2022) exploit the model's attention activations to identify the part of the input attended by the model during the prediction. They can be used to explain predictions in diverse tasks, like image recognition (Li et al., 2021), authorship verification (Boenninghoff et al., 2019) gender bias identification (Boenninghoff et al., 2019) etc. On the other hand, Gradient-based methods (Springenberg et al., 2014; Selvaraju et al., 2017) compute feature attributions by manipulating the gradients computed in the backward step with respect to the original inputs (Shrikumar et al., 2016), or with respect to a specific baseline (Simonyan et al., 2013; Sundararajan et al., 2017).

*Black-box methods* do not make any assumptions regarding the underlying model. For example, Permutation Feature Importance (Breiman, 2001), initially designed for random forests and later extended into a model-agnostic version by Fisher et al. (2019), consists in randomly shuffling the input features and evaluating the model's output variations. Ribeiro et al. (2016) proposed LIME (Local Interpretable Model-Agnostic Explanation), which uses a surrogate linear model to approximate the black-box model locally, that is, in the neighborhood of any prediction. LOCO (Lei et al., 2018) is another popular technique for generating local explanation models. It can provide insight into the importance of individual variables in explaining a specific prediction. SHAP (Lundberg and Lee, 2017) is a framework considered by many to be the gold standard for local explanations, thanks to its solid theoretical foundation. SHAP leverages the concept of Shapley values, first introduced by Shapley et al. (1953), used to measure the contribution of players in a cooperative game. This was later extended by Lundberg and Lee (2017) for the purpose of explaining a machine learning model.

In this work, we propose a flexible hybrid framework based on SHAP, which benefits from properties typical of *black-box* methods, since it can be applied in a completely model-agnostic way. At the same time, our method shares some properties with *white-box* approaches since, when possible, it takes advantage of certain internal components of the model. In particular, the framework we propose for Vision-Language generative models can be leveraged to exploit architectural features of a model's visual backbone to generate more semantically meaningful explanations.

### 2.2. Background on SHAP

In the context of machine learning, the cooperative framework introduced by Shapley et al. (1953) can be framed as a game where each input feature is a player and the outcome is determined by the model's prediction. Shapley values measure the contribution of each player to the final outcome, or in other words, the input features' importance. Shapley redistributed the total outcome value among all the features, based on their marginal contribution across the possible coalitions of players, i.e. combinations of input features. The outcome of the game, namely the prediction of the model, is redistributed across the features, in the form of contributions that have three desirable properties:

- *Efficiency*: all the Shapley values add to the final outcome of the game;
- *Symmetry*: all the features generating the same outcome in the game have the same Shapley value, thus the same contribution;
- *Dummy*: if adding a feature to a coalition (i.e., set of features) does not change the outcome of the game, its Shapley value is zero.

Furthermore, Lundberg and Lee (2017) contribute by formulating a variety of methods to efficiently approximate Shapley values in different conditions:

1. KernelSHAP: derived from LIME and totally model agnostic, hence the slowest within the framework;
2. LinearSHAP: designed specifically for Linear models;
3. DeepSHAP: adapted from DeepLift (Shrikumar et al., 2017) for neural networks, which is faster than KernelSHAP, but makes assumptions about the model's compositional nature.

Later on, the framework was extended with other methods with variations for specific settings; Mosca et al. (2022a) propose a thorough description of the SHAP family of methods.

It is important to note that all these methods work under the so-called *feature independence assumption*, which is fundamental for the theoretical resolution of the problem. Feature independence assumes that no two features are correlated or overlapping. Since Shapley (and SHAP) attributions are computed by marginalizing features, if a feature is strongly correlated to or overlaps with another, such marginalization yields unrealistic results (Molnar, 2020). However, in order to deal with real-life scenarios, this constraint can be relaxed to some extent. For instance, in Natural Language Processing tasks each token of a textual sequence is considered an independent feature (Kokalj et al., 2021) whereas, in Computer Vision, the image is usually split into squared patches or superpixels, which are also considered independent of each other (Jeyakumar et al., 2020). In both of these cases, the independence assumption is a simplification. For example, language tokens are often mutually dependent in context (and this is indeed the property leveraged by self-attention in Transformer language models). Similarly, pixels in neighboring patches in an image may well belong to the same semantically relevant region (and this is indeed the property exploited by neural architectures suited for computer vision tasks, such as convolutional networks). Properties of tokens in context and those of pixels in image regions, have been taken into account in some adaptations of SHAP which consider the hierarchical structure of the feature space, such as HEDGE for text (Chen et al., 2020) and h-SHAP for images (Teneggi et al., 2022).

In our work, we also relax the independence assumption, an issue we discuss in detail in Section 4.4.2.

### 2.3. Kernel shap

The core method of our framework is Kernel Shap. We base our approach on the formulation by Lundberg and Lee (2017), which provides an accurate regression-based, model-agnostic estimation of Shapley values. The computation is performed by estimating the parameters of an explanation model *g*(*x*′) which matches the original model *f*(*x*), namely:


(1) $$\begin{array}{l} f\left(x\right)=g\left(x^{\prime }\right)=\phi_{0}+\overset{M}{\underset{i=1}{\sum }}\phi_{i}x_{i}^{\prime } \end{array}$$

where *M* is the number of input features (or players) and $x_{i}^{\prime }$ is a player of the game. *g*(*x*′) is approximated by performing a weighted linear regression using the Shapley kernel:


(2) $$\begin{array}{l} \pi_{x^{\prime }}\left(z^{\prime }\right)=\frac{M-1}{\left(M\;\text{choose }|z^{\prime }|\right)|z^{\prime }|\left(M-|z^{\prime }|\right)} \end{array}$$

where *z*′ is the subset of non-zero entries, namely a binary representation of the coalition of players. The Shapley kernel, in other words, is a function assigning a weight to each coalition. The number of coalitions needed to approximate the Shapley values corresponds to all the possible combinations of players, i.e., 2^*M*^ coalitions. This makes Kernel SHAP extremely expensive to compute (and slow in practice) when *M* is large.

Our framework relies on KernelSHAP as is it totally model-agnostic. We address both the efficiency issue and the strict independence assumption of the method by generating semantic input features (more details in Section 3.2.2) and optimizing the approximation through sampling (full details in Section 3.1).

### 2.4. Explainability for vision and language

One way to characterize the scope of V&L models is with respect to the types of tasks they are designed to address. On the one hand, tasks like image captioning (Anderson et al., 2018; Fisch et al., 2020; Mokady et al., 2021; Zhang et al., 2021; Li et al., 2022a), image-text retrieval (Radford et al., 2021; Cao et al., 2022), and visual question answering (Antol et al., 2015) require a strong focus on the recognition of objects in images. More recently, research has begun to explore the capabilities of models in tasks that require some further reasoning or inference over the image contexts, such as understanding analogies (Zhang et al., 2019a), describing actions and rationales (Cafagna et al., 2023) and inferring temporal relations (Park et al., 2020).

The need to understand how V&L models ground their predictions has become essential, leading to the emergence of Explainable Artificial Intelligence (XAI) for multimodal settings (Zellers et al., 2019). Visual explanations can help humans to know what triggered the system's output and how the system attended to the image. To this purpose, feature attribution methods are often preferred as they can provide a visual explanation of the prediction. Most of the XAI methods introduced for unimodal tasks can be adapted to V&L tasks.

Some popular *white-box* methods use gradients to generate saliency maps to highlight the pixels corresponding to highly contributing regions. These methods include Grad-CAM (Shrikumar et al., 2016; Selvaraju et al., 2017) or Layer-wise Relevance Propagation (LRP) (Binder et al., 2016) where the contribution is computed with respect to an intermediate layer instead of the input layer. These methods can produce fine-grained pixel-level explanations. However, their outcomes can be noisy and require many evaluations to converge to a stable explanation.

*Black-box* approaches are mostly perturbation-based, that is, they compute attributions based on the difference observed in the model's prediction by altering the input. Such methods include occlusion sensitivity, RISE (Petsiuk et al., 2018), and LIME (Ribeiro et al., 2016). Other approaches are task-agnostic, like MM-SHAP (Parcalabescu and Frank, 2022), where a SHAP-based method is used to measure the contribution of the two modalities in V&L models independently of the task performance. Although these methods make few assumptions about the underlying model, their explanations are computationally expensive, as the number of model evaluations required grows exponentially with the number of features. To overcome this limitation, the number of features is usually reduced by partitioning the image into patches called superpixels, which discretize the input into a smaller number of features. However, this approach can lead to coarser and not very informative explanations.

Explanations for V&L **generative tasks**, like image captioning, incur even more complexity, as the prediction of the model is now a textual sequence. As noted in Section 2.2, SHAP estimates feature contributions based on the amount of variation observed in the model output, with or without the feature. This requires a numerical output value (which of course, linguistic sequences are not). A popular solution, which is in keeping with the autoregressive nature of neural language decoders, is to break down the caption generation process into a series of steps where each token is explained separately with respect to the image and the previously generated sequence. This requires generating a single visual explanation for each generation step. However, the meaning of the sentence is not only determined by the meaning of the single words it is composed of but also by the way these words are combined and arranged together. Therefore, a global meaningful explanation must take into account the whole textual sequence and not just part of it, as only in this way can the explanation take into account the whole textual context.

A popular solution is to generate the token-level explanations using Integrated Gradients (Sundararajan et al., 2017), providing region-level visualizations or using the attention activation scores to visualize the model's attended regions (Zhang et al., 2019b; Cornia et al., 2022). However, these methods are white-box approaches as they make assumptions about the inner workings of the model; thus they need to be specifically re-adapted to new systems. Furthermore, they focus on token-level explanations but do not allow a comprehensive global explanation of the textual output.

To the best of our knowledge, our work is the first attempt to bring together a model-agnostic framework like SHAP, in the image-to-text task, with the aim of providing a comprehensive explanation of the generated textual output as a whole, rather than on a token-by-token level. We further propose a method to provide explanations based on features that are semantically meaningful, rather than on patches or superpixels.

## 3. Method

In this section, we first address the matter of efficiency which, as noted above, is a pressing problem for methods based on Kernel SHAP. We then turn to the core proposals in our method, adapting it to generative models to achieve explanations for whole sequences rather than tokens (Section 3.2.1) and using semantically meaningful visual regions as features (Section 3.2.2).

### 3.1. Kernel SHAP sampling

Kernel SHAP is model-agnostic, meaning that it cannot make any assumption on the model to explain. For this reason, it is also among the slowest in the SHAP family of XAI methods (Mosca et al., 2022b). This issue is addressed by performing Monte Carlo sampling over the pool of coalitions, allowing under certain conditions to compute a reasonably accurate approximation of the Shapley values, even in the case of large-sized models or low-resource hardware.

**Figure 1:**
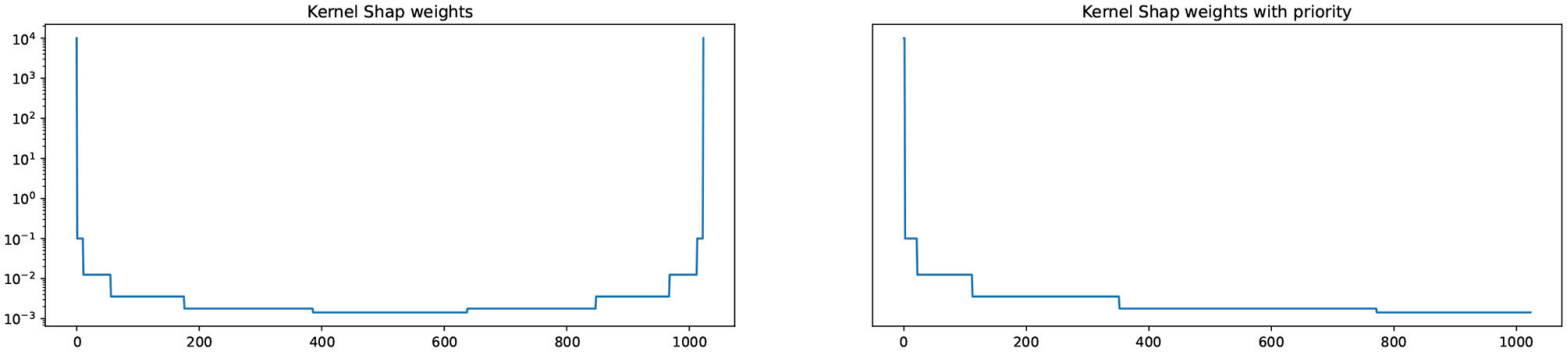
Standard Kernel SHAP **(left)** and modified Kernel SHAP with priority for high-weight coalitions **(right)**. The y-axis corresponds to weight whereas the x-axis is the iteration in which a particular coalition is generated.

Taking inspiration from Molnar (2020), we implement a deterministic sampling strategy. Given a specific sampling budget *k*, we prioritize coalitions which have a high weight, where weight is computed by Equation 2. This is achieved by generating the coalitions in decreasing weight order and selecting the first *k* coalitions. In Figure 1 we compare the weights of coalitions computed using the standard Kernel SHAP (on the left) and using the method which prioritizes high-weight coalitions (on the right). As can be observed, our sampling strategy with priority (on the right) ensures that we select the high-weight coalitions first, providing an optimal ordering among samples.

Sampling with priority offers two main advantages:

1. higher accuracy of the Shapley values estimate;
2. a deterministic sampling strategy.

**Figure 2:**
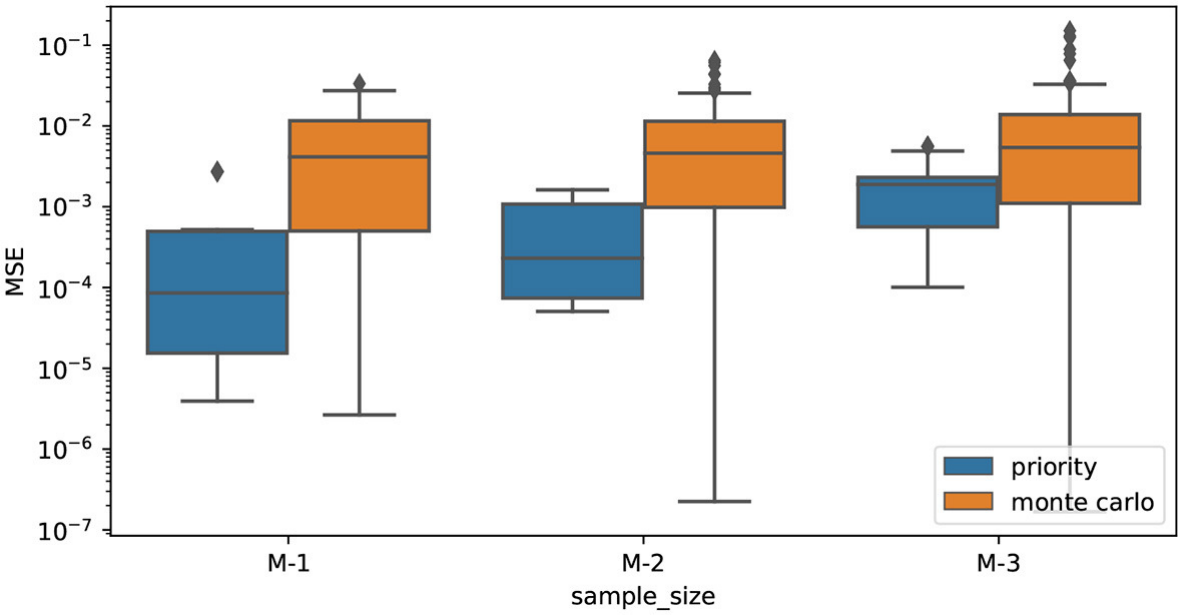
Mean Squared Error (MSE) of the Shapley values estimated using Monte Carlo sampling (orange) and sampling coalitions with priority (blue), for various sampling sizes. All the values on the x-axis are exponentials (2^*M*−1^, 2^*M*−2^, 2^*M*−3^) where *M* corresponds to the number of features. The MSE is computed with respect to the Shapley values computed using all the 2^*M*^ coalitions available in the sampling space.

In Figure 2 we report the approximation error of the Shapley values when applying Kernel SHAP, using Monte Carlo (orange) and the high-weight priority (blue) as sampling strategies, for different sample sizes. The error is computed over 10 runs, using the Mean Squared Error (MSE) with respect to the Shapley values computed with Kernel SHAP using all the 2^*M*^ coalitions. Our sampling with priority approximates Shapley values with errors that are orders of magnitude smaller than Monte Carlo sampling. We observe this consistently for different sampling sizes.

With a more efficient and deterministic sampling strategy, we now turn to the core of our method.

### 3.2. Adapting Kernel SHAP to vision and language generative tasks

In the image captioning scenario, we can set up a cooperative game, where we want to compute the contributions of the players, i.e. the pixels of the image, with respect to the outcome, i.e., the caption. In Section 2, we identified two shortcomings of the standard way in which this is performed. Here, we discuss our contributions to overcome these shortcomings.

The first problem is related to the comprehensiveness of explanations. In order to measure the variations of the outcome of the function needed to run Kernel SHAP, the caption generation process is usually broken down into token generation steps. Each step produces logits that can be used to compute a numerical outcome. However, this forces us to consider each generation step as a separate cooperative game, meaning that we need to run a separate instance of Kernel SHAP for each generated token, further increasing the time and compute cost needed to explain an image-caption pair. Moreover, such explanations refer to single tokens and do not provide an explanation for the whole output of the model, namely the caption.

The second problem is related to the definition of coalitions in the visual input. The number of coalitions to be computed grows exponentially with the number of players. This makes the computation of the Shapley values intractable for images and makes any sampling strategy inaccurate. In order to overcome this limitation, the image is typically partitioned into a grid composed of *superpixels*, namely groups of pixels, each of which represents a single player. This reduces the total number of players in the game, making computation of the Shapley values more feasible, but at the same time, it reduces the degree of the detail of the explanation. Moreover, we argue that breaking the image into a grid of square superpixels breaks the semantics underlying the image, resulting in potentially under-informative explanations. In particular, there is no guarantee that the pixels grouped together in this manner correspond to semantically meaningful image regions.

**Figure 3:**
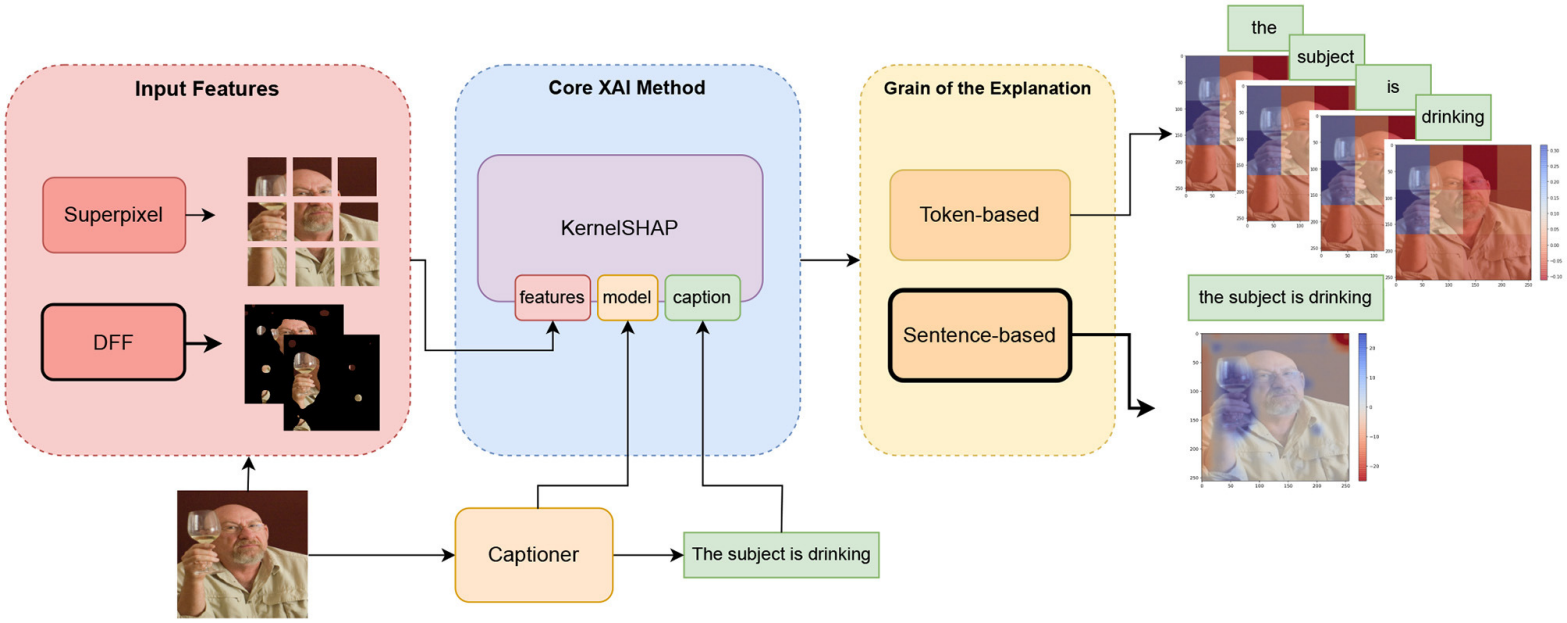
Overview of the explainability framework. The new components proposed in this work are shown with a dark border. Our method leverages **KernelSHAP** as the core explainability method. We introduce semantic features extracted using **DFF** from the captioner's visual backbone and generate **sentence-based** visual explanations based on the estimated Shapley values.

In the following sections, we address these issues, proposing alternative solutions. Specifically, we address the first shortcoming in Section 3.2.1, before turning to a proposal for semantically meaningful and sparse priors in Section 3.2.2. Our solution can be integrated with existing methods, to compose a modular explainability framework for generative V&L models. An overview of this framework is shown in Figure 3.

#### 3.2.1. Toward sentence-based explanations

In order to adapt Kernel SHAP to generate global explanations for the caption, we measure variations of the caption's meaning representation when perturbations are applied to the input image. This allows us to numerically quantify the meaning variation of the whole caption which is due to the marginal contributions of different input features (image regions or pixels).

Formally, given an image-captioning model *f* and an image *x* we generate a caption *c* = *f*(*x*) and we compute:


(3) $$\begin{array}{l} e_{ref}=E\left(c\right) \end{array}$$

where *e*_*ref*_ is the embedding representation of *c* that we consider the *reference embedding* of the caption, and *E*() is a function used to extract such a representation.

For each perturbed image *x*′ and its corresponding caption we extract, analogously, an embedding *e*′. Then we compute:


(4) $$\begin{array}{l} s=cos\left(e_{ref},e^{\prime }\right) \end{array}$$

where *s* is the variation in the embedding representation computed as the cosine distance *cos*(·), between the reference embedding *e*_*ref*_ and the embedding of the caption of the perturbed image *e*′.

**Figure 4:**
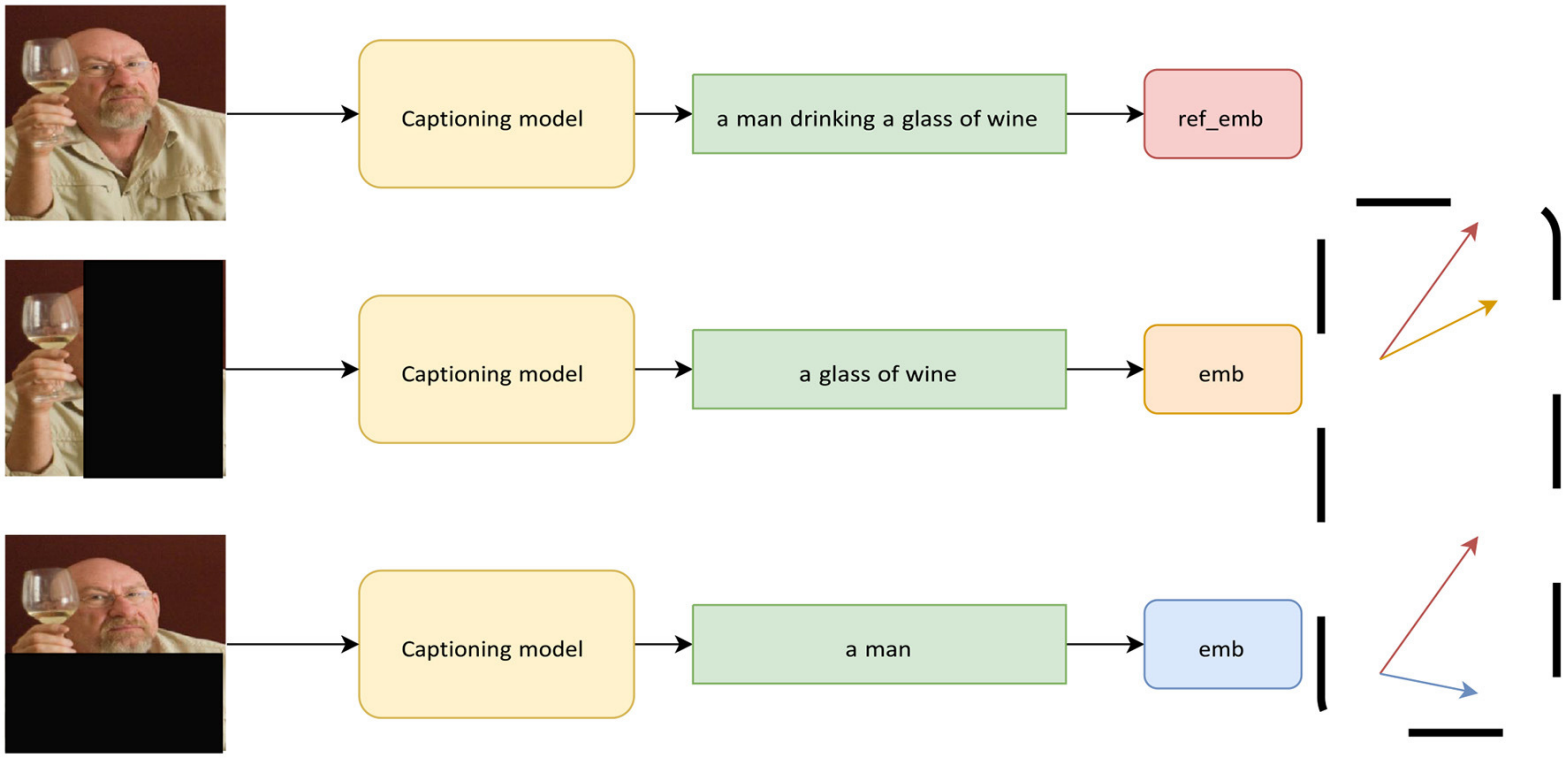
Example of the sentence-based explanation. We compute the reference embedding (red) from the caption generated by the model when the input has no perturbation. For each perturbation applied, we compute the embedding (orange, blue) of the resulting caption and use the cosine distance between the reference and the current embedding, to measure the semantic variation of the caption.

In other words, we use the cosine distance between the semantic representation of the reference caption and the caption generated upon input perturbation, to measure the model's variations. A schematic representation of the method is shown in Figure 4.

Re-framing the problem as described allows us to apply Kernel SHAP to compute feature attributions taking into account the semantic variation of the whole caption in a single cooperative game instance.

#### 3.2.2. Exploiting semantic visual priors

Partitioning the image into a grid of superpixels is a straightforward way to reduce the number of input features in the image. We argue that, although convenient, superpixels do not guarantee the preservation of semantic information depicting the visual content, as they shatter the image into equally sized patches regardless of the content represented. We address this issue by proposing a semantically guided approach, that selects the input features according to semantics-preserving visual concepts arising from the visual backbone of the V&L model. This not only allows for generating more meaningful explanations but explicitly focuses explanations of the model's generative choices on the output of the model's own visual backbone.

We generate input features leveraging the Deep Feature Factorization (DFF) method (Collins et al., 2018). DFF is an unsupervised method allowing concept discovery from the feature space of CNN-based models. We refer to such concepts as “semantic priors,” that is, the knowledge or assumptions learned by the visual backbone, in the context of a given domain or task. We use them to craft input features that produce semantically informed visual explanations.

Formally, following Collins et al. (2018)'s notation, given the activation tensor for an image *I*:


(5) $$\begin{array}{l} A\in {\mathbb{R}}^{h\times w\times c} \end{array}$$

where *h, w, c* correspond respectively to the height and width, and the number of channels of the visual backbone's last activation layer, we perform a non-negative matrix factorization (NMF) of *A*:


(6) $$\begin{array}{l} NMF(A,k)=\underset{{\hat{A_{I}}}_{k}}{\text{argmin}}\;\|A-{\hat{A}}_{k}{\|}_{F}^{2},\\ \text{ subject to }{\hat{A}}_{K}=HW,\\ \;\forall i,j:H_{ij},W_{ij}\geq 0, \end{array}$$

where *W*∈ℝ^*n*×*k*^ and *H*∈ℝ^*k*×*m*^ enforce the dimensionality reduction to rank *k*.

**Figure 5:**
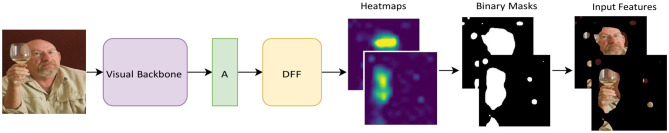
Schematic example of input features extraction using DFF. Through thresholding, we convert the heatmaps into binary masks that we use to create semantically meaningful features.

Each column *H*_*j*_ can be reshaped into *k* heatmaps of dimensions *h*×*w*, each of which highlights a region that the factor *W*_*j*_ corresponds to. The heatmaps are then upsampled to match the original image size with bilinear interpolation and converted to binary masks, each of which corresponds to an input feature. In this way we obtain *k* input features, where *k* is the number of concepts extracted. A schematic example of input feature extraction performed by DFF is shown in Figure 5.

In our method, the regions identified via DFF are the features for which attributions are computed. The key intuition is that these features correspond to meaningful sub-parts of the input image according to the V&L model's visual backbone. They do not necessarily reflect humans' visual expectations of the image (although we find that they often do); rather they represent the visual priors learned by the vision model after training.

To create a coalition we sum up multiple masks, then apply them to the original image, which will contain only pixels belonging to input features in the selected coalition.

NMF can be seen as an unsupervised clustering algorithm, allowing control for the number of clusters or concepts to find. *k* can be considered a hyperparameter of the method, which we show can be kept small to achieve a good level of semantic detail and low compute cost.

##### 3.2.2.1. Non-partitioning features

DFF generates semantic masks reflecting the activations of the model's visual backbone. The whole process is unsupervised and produces masks that do not constitute partitions of the image, meaning that it is not guaranteed that the sum of all the extracted masks will match the total size of the image.

In order to account for this issue, we create an additional *leftover* mask covering the remaining area and we include it in the SHAP cooperative game, this allows us to consider the whole visual information represented by the image, in the game.

As noted in Section 2.2, the computation of Shapley values is based on a feature independence assumption. Since our features may be non-partitioning, this constraint may not hold, thus we relax this assumption in our approach. We explore the consequences of this in more detail in Section 4.4.2.

##### 3.2.2.2. Intensity-preserving explanations

SHAP-based methods relying on superpixels assume that each pixel in a patch contributes equally, thus all the pixels in a patch are assigned the same Shapley value. However, in DFF, features in each binary mask correspond to an equally-sized heatmap. Therefore, we multiply the Shapley value by the heatmap corresponding to the binary mask. This allows scaling the contribution according to the intensity of the feature signal.

## 4. Experiments

The methodology described in the previous section raises an important question which we now address experimentally: *What are the pros and cons of our method based on visual semantic priors in comparison with standard feature selection methods used in V&L , based on superpixels?*

In this section, we describe the data and task, as well as a state-of-the-art vision-to-language model, which we used to perform a human evaluation of our explainability framework.

### 4.1. Data

We validate the method presented in the previous section with experiments using the HL image caption dataset. The HL dataset (Cafagna et al., 2023) contains 15k images extracted from COCO (Lin et al., 2014). The dataset pairs images with captions that describe the visual contents along three different high-level dimensions, namely *scenes, actions* and *rationales* for the actions. These are additionally paired with the original COCO captions, which provide a more low-level, object-centric description. The annotations were collected by asking annotators three questions related to each of the three high-level dimensions. The systematic alignment of the object-centric and abstract captions provides us with a suitable test bed to compare the efficacy of our method in delivering global explanations in both captioning and visual question-answering scenarios. An example pairing the three high-level captions and the original COCO caption from the HL Dataset is shown in the Supplementary material.

### 4.2. Model

For our experiments, we focus on one V&L model, since our goal is to evaluate the quality of explanations, not the model itself. Our choice is motivated by two considerations: first, a model should ideally have good performance in zero-shot settings; second, it should exhibit state-of-the-art performance on generative tasks. OFA (Wang et al., 2022) is a large pre-trained multimodal model with a CNN-based visual backbone, trained using a task-agnostic and modality-agnostic framework. OFA is able to perform a diverse set of cross-modal and unimodal tasks, like image captioning, visual question answering, image generation, image classification, etc. OFA is trained on a relatively small amount of data (20M image-text pairs) with instruction-based learning and a simple sequence-to-sequence architecture. Nevertheless, on downstream tasks, it outperforms or is on par with larger models trained on a larger amount of data. OFA is effectively able to transfer to unseen tasks and domains in zero-shot settings, proving to be well grounded also in out-of-domain tasks.

This makes OFA an excellent candidate to test our explainability framework in a real-world scenario, namely a large pre-trained generative model with SOTA performance on downstream tasks in zero-shot conditions. Thus we use OFA to generate textual predictions in a VQA setting. We then use our framework, which combines DFF features and sentence-based explanations, to generate visual explanations of such predictions. In our evaluation, we compare these explanations to the more standard setup for vision-to-text XAI, one based on superpixels as features.

### 4.3. DFF vs Superpixel

In this Section, we focus on the comparison between the global visual explanations produced using superpixel or DFF input features. We focus on the capability of the two methods to adapt to different semantic aspects of the explanation; in Section 4.3.1 we specifically address this discussion with a focus on the VQA task.

All the experiments are performed in zero-shot by using the *OFA-large* model in its original implementation.^1^ In order to ensure a fair comparison, we extract a similar number of features for both methods, namely 12 for superpixel and 11 for DFF. This number allows us to execute the experiments in a reasonable amount of time. In fact, we recall that the number of features has an exponential impact on the number of model evaluations needed to generate the explanations. Reducing the number of features mitigates the efficiency issue, but does not solve it. An in-depth discussion about the efficiency issue is provided in Section 4.3.2.

**Figure 6:**
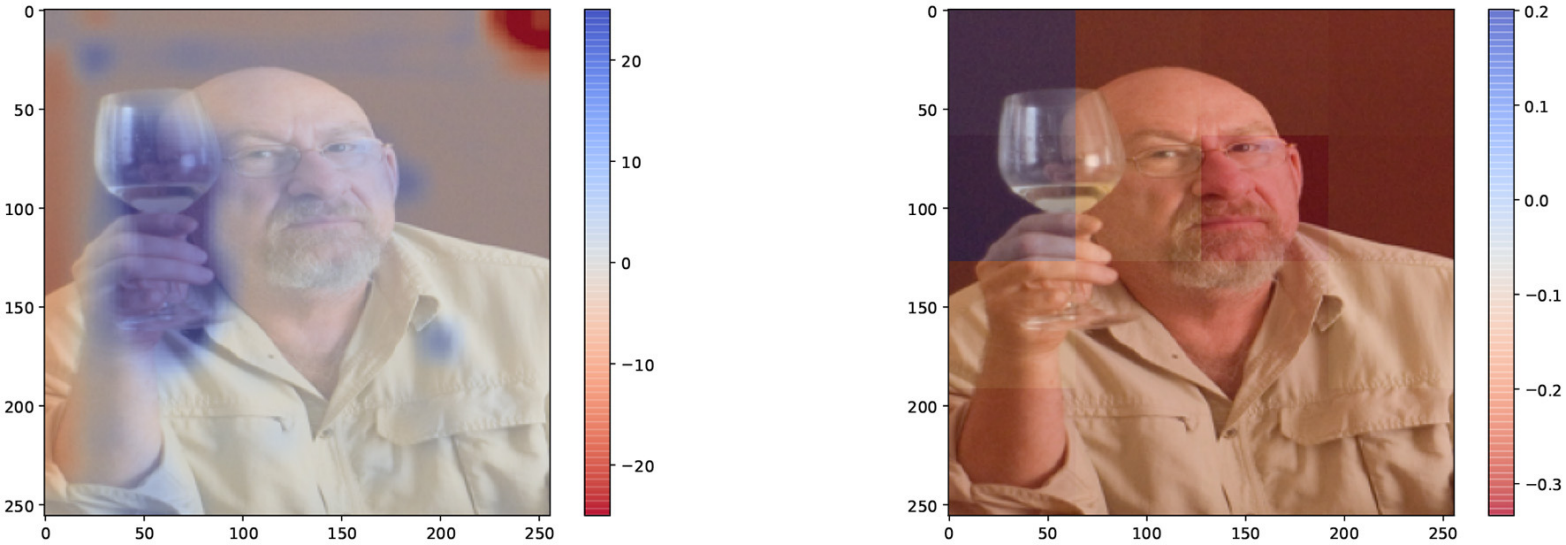
Global visual explanation for the question “What is the subject doing?,” and corresponding model's answer “drinking.” Explanations are generated using Kernel SHAP. The explanation using DFF input features **(left)** provides a detailed positive (blue) area. We use 11 DFF-features and 12 superpixel-features. The explanation generated by superpixel input features **(right)** although covering a similar region, i.e., the glass, does not provide the same level of detail.

As an initial comparison, Figure 6 shows a direct comparison between the two kinds of input features for the caption “drinking,” generated using sentence-based Kernel SHAP. Both methods assign a positive contribution to the region corresponding to the glass, with some important differences:

- **Detail**: The DFF features succeed in capturing the key visual semantics of the image, i.e., the glass, in a single input feature (with some noise), producing a more detailed explanation than superpixel, where the region corresponding to the glass is shared across different patches (i.e., different features).
- **Intensity**: DFF scales the contributions according to the magnitude of the feature signal (as described in Section 3.2.2.2), providing a fine-grained visual indication of the importance of specific sub-regions within the same input feature region.

#### 4.3.1. Semantic visual features improve the quality of the explanations

We compare DFF and superpixel explanations on the VQA task. We select images and questions for the three axes in the HL dataset, i.e., action, scenes, and rationales, and we generate visual explanations for the answers. This allows us to compare how the two methods handle semantically different aspects highlighted in the visual content.

**Figure 7:**
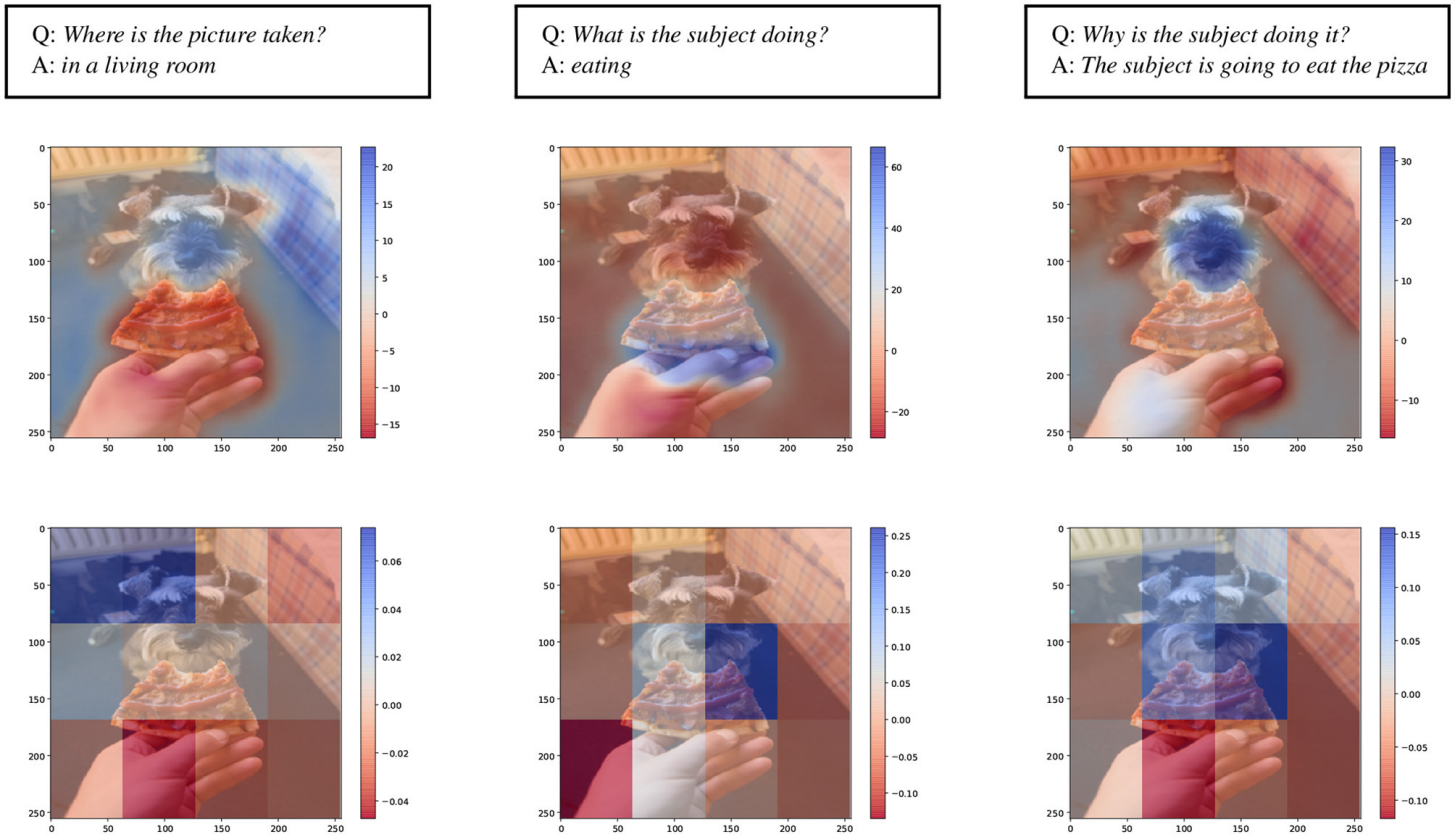
Examples of explanations for the VQA task from the HL Dataset for the *scene, action* and *rationale* axes. In the top row are shown the questions **(Q)** and the generated answers **(A)**. The middle and the bottom row, show visual explanation generated respectively with DFF and superpixel input features, with comparable compute cost.

We expect to see that the positive contribution assignment (in blue) changes for the same image for different captions, corresponding to different kinds of questions for which the model generates different answers. In response to different questions about location, rationale, or action, the model's output should depend on different regions of the image. For instance, we expect to observe a wider positive area highlighted in the picture for the *where* question and a more specific detailed area for the *what* question. As shown in Figure 7, the DFF-based method (middle row) succeeds in highlighting in significant detail the semantic areas contributing to the output. On the other hand, superpixels provide coarser detail, as they are limited by the size of the patches. This suggests that the DFF-generated explanations could lead to a visible advantage in terms of comprehensiveness and completeness; we further test these hypotheses by running a human evaluation, in Section 6.

#### 4.3.2. Semantics-guided explanations are efficient

**Figure 8:**
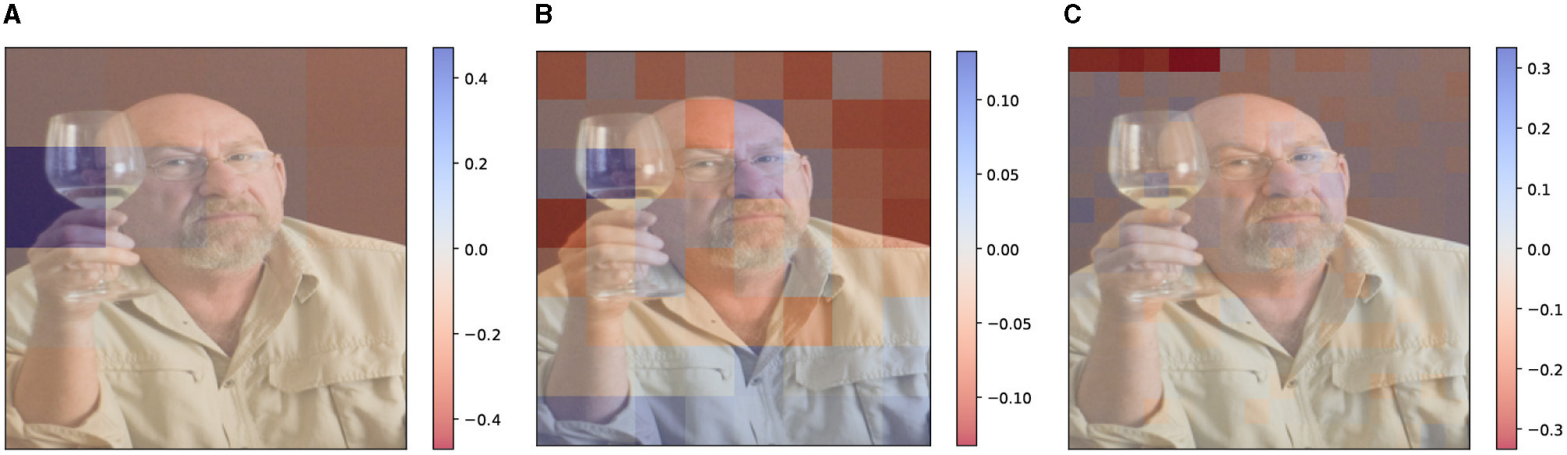
Example of explanations generated with superpixels, with an increasing number of features, namely 16, 64, 256 features [respectively **(A–C)**]. These are obtained with Kernel Shap sampling using a fixed sampling budget of 2, 048 samples. **(A)** 4X4. **(B)** 8x8. **(C)** 16x16.

In order for superpixel-based explanations to achieve a level of detail comparable to DFF, we need to significantly increase the number of patches. However, this causes an exponential surge in computing cost, which makes it unfeasible to run, especially if we are testing large models. This issue can be mitigated by performing Kernel SHAP sampling (as described in Section 3.1). The combination of the exponential growth of the sample space, and the limited sampling budget can easily lead to unreliable explanations. An example is shown in Figure 8 where we perform Kernel SHAP sampling with a budget of 2048 samples, which is the same budget used to compute the DFF explanation in Figure 6.

On the other hand, DFF does not suffer from this issue. In fact, there is no clear advantage in increasing the number of features, because the main semantic content is already embedded in a small number of features. In our experiments, we establish that a good number of features for DFF is between 8 and 12. This number of features keeps the computational cost low, allowing us to compute full Kernel SHAP or Kernel SHAP sampling with very high accuracy. We provide full details in the Supplementary material.

### 4.4. Semantic features analysis

The semantic features extracted by DFF are drastically different from superpixel features in many key aspects related to the visual content captured. Moreover, DFF is unsupervised and dynamically exploits the visual backbone's priors. In this Section, we focus on analyzing the benefits and limitations characterizing the semantic features generated by DFF. We discuss in detail key aspects like the kind of semantic content captured along with possible theoretical implications and how it can be generalized over different visual backbones.

#### 4.4.1. What kind of semantics do DFF features capture?

**Figure 9:**
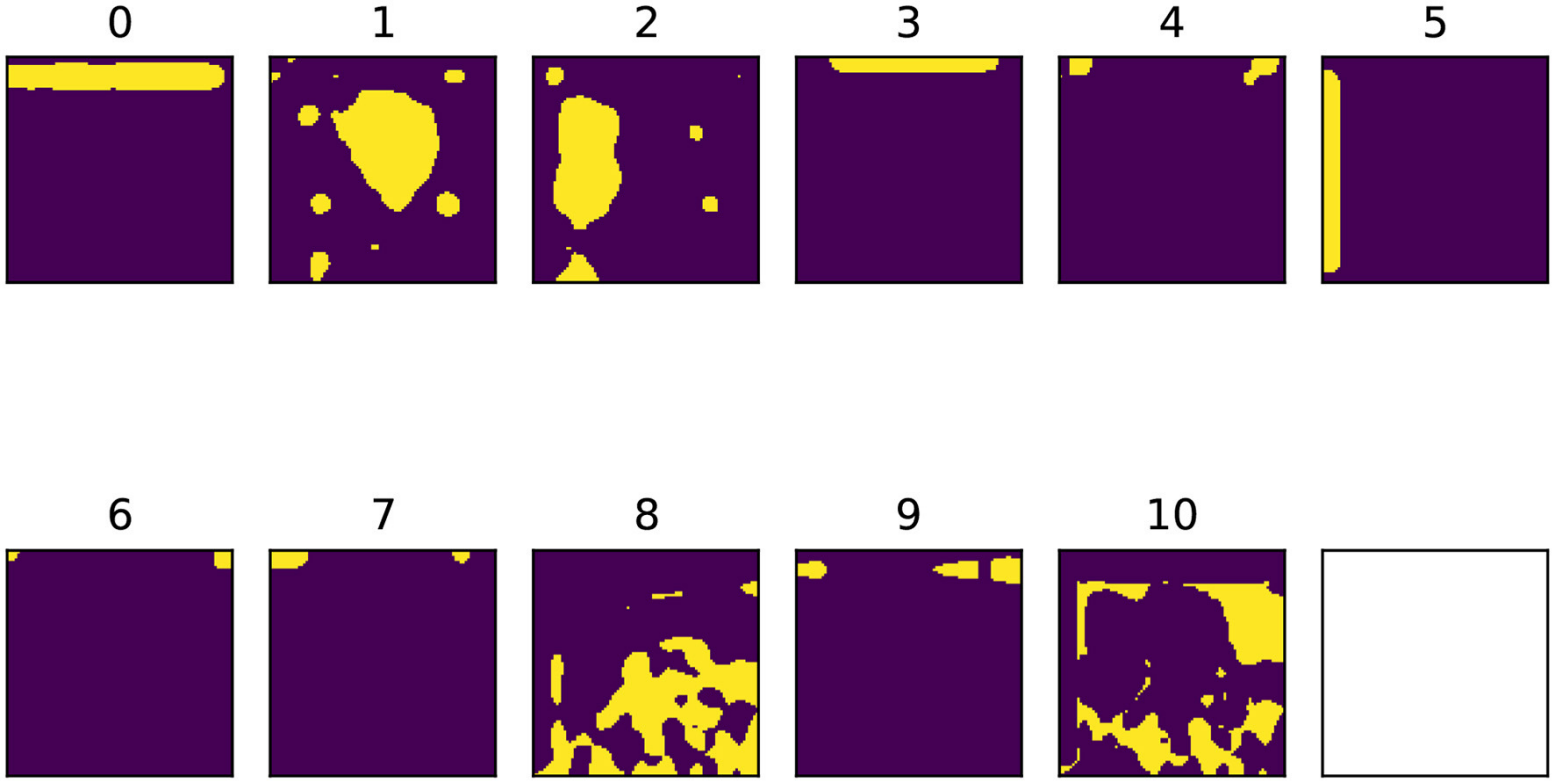
Binary feature masks extracted using DFF with *k* = 10. The 11^*th*^ feature is the *leftover* mask. The original image is the same shown in Figures 6, 8.

DFF features capture semantic concepts learned by the model's visual backbone. These do not necessarily follow human visual expectations. In Figure 9 we show an example: features 1, 2, and 8 can be associated with three main **semantic objects and entities** of the image, namely *face, glass* and *shirt*. However, we observe in the remaining features **several geometrical patterns**, that highlight the edges and the corners of the pictures. This pattern is recurrent in the features extracted by DFF, independently of the visual content. We believe this is partially due to the capability of CNNs to capture spatial configuration (Zeiler and Fergus, 2014) and the effectiveness of DFF in factorizing together model activations with similar characteristics.

#### 4.4.2. Relaxing the feature independence assumption

As described in Section 2.2, SHAP in the cooperative game formulation assumes the *feature independence principle*, namely that each feature is independent of all the others. However, this assumption does not hold for image data since each pixel is inherently dependent on the other pixels, especially those in its vicinity. Therefore, in order to work with visual data, this constraint needs to be relaxed. This solution is typically applied for computer vision tasks by graphical models like Conditional Random Fields (CRF). CRFs relax the strong dependence assumption on the observations (the pixels of the image) by modeling the joint distribution of observations, usually intractable, as a conditional distribution (Li et al., 2022b).

Along the same lines, superpixel features relax this constraint by partitioning the image into patches that are not independent, considering the underlying semantics depicted in the visual content.

**Figure 10:**
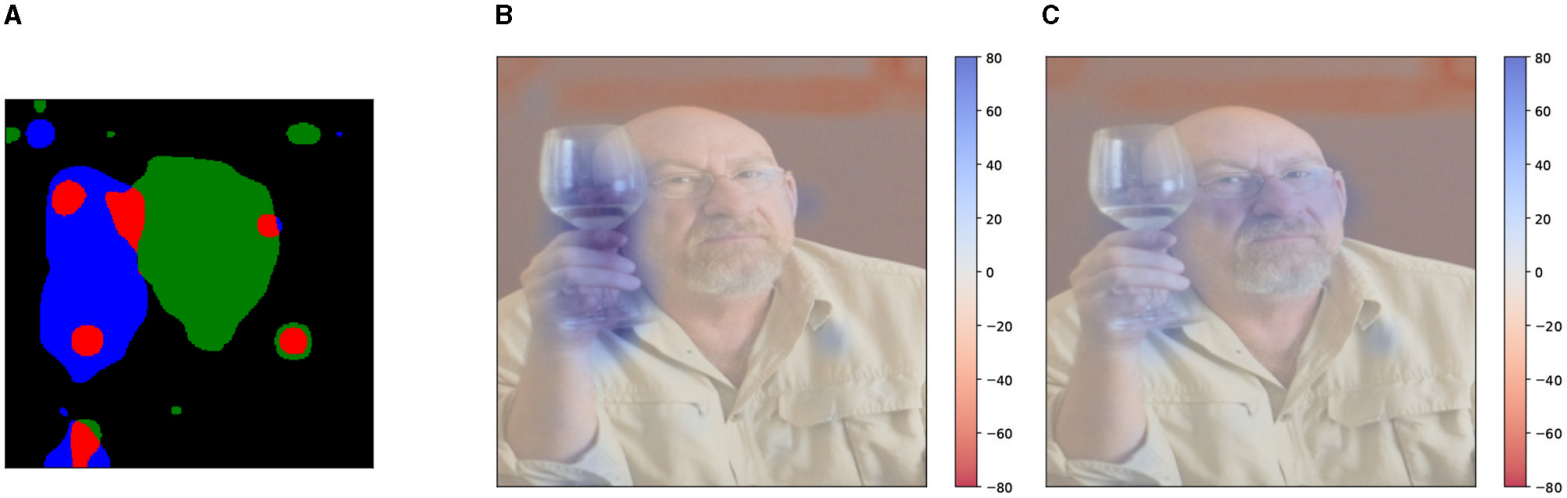
Example of overlap (highlighted in red) between two feature masks **(A)** and comparison between visual explanations generated given the question “What is the subject doing?” and the model's answer “drinking.” We compare regular DFF features **(B)** and disjoint DFF features **(C)**. Although the masks overlap only to a small extent, the explanation is visibly affected. **(A)** Overlapping features. **(B)** Non-disjoint features. **(C)** Disjoint features.

This issue is mitigated by the DFF features, as they tend to cover semantically related regions of the image, preserving the underlying visual semantics. On the other hand, as pointed out in Section 3.2.2.1, DFF features are not disjoint, meaning that to some extent, the contribution of overlapping regions is subject to contamination from other regions. In this section, we analyse the consequences of this in more detail. Our analysis follows two steps:

1. We measure the DFF feature overlap over a sample of 1,000 images. We find that the amount of overlap among the feature masks corresponds to 0.77% of the pixels in the image with a standard deviation of 0.63 and an average maximum peak of 2.04%. This suggests that this phenomenon is present to a limited extent, at least for the model we are using.
2. We compare visual explanations generated by disjoint and non-disjoint features. In order to generate disjoint features, we post-process the feature masks extracted, by checking all possible pairs of feature masks and assigning the possible overlapping region to one of the two compared features. An example is shown in Figure 10A where the overlapping regions (highlighted in red) between two feature masks are randomly assigned to one of the features (either blue or green).

Enforcing the features' disjointness leads to similar results to their non-disjoint counterpart. However, in some cases, the re-allocation of the overlapped region impacts the Shapley value of the feature, causing unpredictable results. This suggests that manually changing the feature masks can disruptively affect the visual semantics captured by the feature, leading to misleading visual explanations. A cherry-picked example is shown in Figure 10, where using the disjoint features (Figure 10C) causes a meaningful change in the visual explanation.

In conclusion, we observe that **the phenomenon of non-disjoint features is present to a limited extent** and overall **it does not invalidate the visual explanations**, as it can be considered a relaxation of the feature independence assumption. Moreover, as empirically observed, relaxing this assumption is unlikely to invalidate the method, as the explanation is consistent with the ones generated by superpixel features. On the other hand, we observed that **forcing the feature masks' disjointness harms their capability to preserve the visual semantics, leading to misleading visual explanations**.

#### 4.4.3. Does feature size matter?

Differently from superpixel patches, DFF semantic features can have different sizes, depending on the semantic role of the highlighted region. We ask to what extent the size of a visual feature could affect the final contribution in the SHAP cooperative game. In order to test for that, we normalize the Shapley value obtained according to the size of the feature mask and we compare normalized values with the un-normalized ones. To normalize a Shapley value we compute:


(7a) $$r_{i}=\frac{\sum_{j=0}^{\left|M_{i}\right|}m_{j}}{\left|M_{i}\right|}$$

(7b) $${\hat{a}}_{i}=\frac{a_{i}}{r_{i}}$$

where *m*_*j*_ is a non-zero element of the binary mask, |*M*_*i*_| is the total number of entries in mask *i* and *r*_*i*_ indicates the proportion of the image covered by the mask. *r*_*i*_ is then used to discount the magnitude of the Shapley value *a*_*i*_ obtaining the normalized value â_*i*_.

In the normalization process, the feature contribution's magnitude is obviously re-scaled. However, we are interested in measuring to what extent the normalization has affected the features' importance in relative terms. Therefore, we use the Rank Biased Overlap (RBO) (Webber et al., 2010), a similarity metric for ranked lists, to measure the difference in the feature attribution ranking after normalization for a sample of 100 DFF-based explanations. A significant change in feature ranking would entail a positive correlation between size and feature importance.

**Figure 11:**
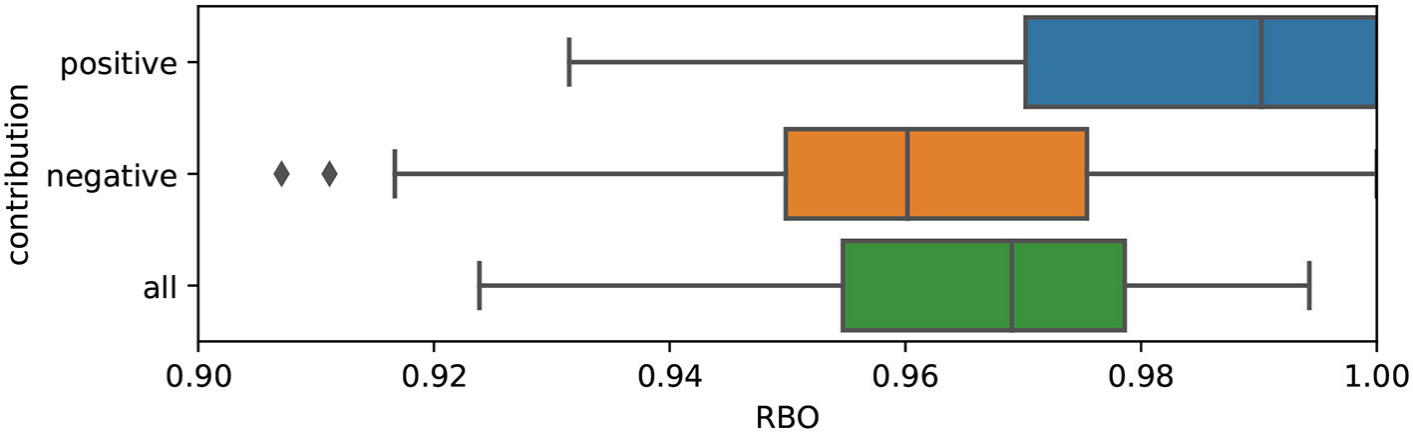
RBO scores computed between normalized and un-normalized Shapley values, for positive (blue), negative (orange), and all (green) features.

In Figure 11 we show the results of this experiment: the RBO is overall at ceiling, with a minimum value of 0.9 (in a range where 1 is identical ranking and 0 is totally different). The positive contributions, which are the most informative to understand the explanations, are the most stable in terms of ranking. This suggests that **the size of the features extracted using DFF does not significantly affect the final contribution of the semantic features and does not harm the visual explanations**.

### 4.5. Does DFF adapt to other visual backbones?

DFF is designed to perform concept discovery in CNN-based visual backbones. However, current pre-trained V&L models' vision encoders often rely on different architectures, such as Vision Transformers (ViT) (Dosovitskiy et al., 2020), FasterRCNNs (FRCNN) (Ren et al., 2015), or their variants. In this section, we show how DFF can be adapted to these architectures. Moreover, we provide an alternative solution to perform model-agnostic semantic feature extraction, which is applicable to any architecture.

#### 4.5.1. Vision transformers

In order to apply DFF to ViT encodings, we need to take into account two substantial differences with respect to CNNs: (1) firstly, ViT splits the image into a grid of patches and generates an embedding vector for each patch. To obtain an activation matrix, each embedding vector is stacked together and a special vector is added in position 0 to indicate the beginning of the sequence. Differently from CNNs, the spatial information related to a patch is lost in the encoding process and added later on, by concatenating a positional embedding to the embedding vectors. (2) Secondly, ViT activations contain both positive and negative values, differently from CNNs which generate only positive activations.

As described in Section 3.2.2, DFF requires a non-negative activation matrix as it is based on NMF, therefore in order to address (2) we normalize the ViT features to values between 0 and 1.

**Figure 12:**
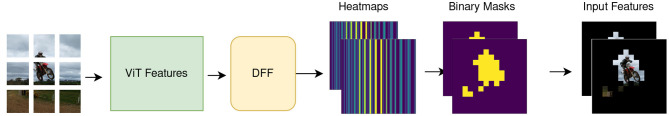
Schematic example of how to generate semantic features with DFF from a ViT visual backbone. The index of the highlighted band in the heatmap is used to select the patches to create the feature.

As a consequence of (1) above, when we apply DFF to the normalized ViT activations, we obtain binary masks with vertical bands, where each band corresponds to a patch in the image. We use the index of the highlighted vectors in the binary mask to select the patches to be grouped together in the semantic features. In this way, **we obtain feature masks by grouping together semantically related patches**. A schematic example is depicted in Figure 12.

#### 4.5.2. FasterRCNNs

**Figure 13:**
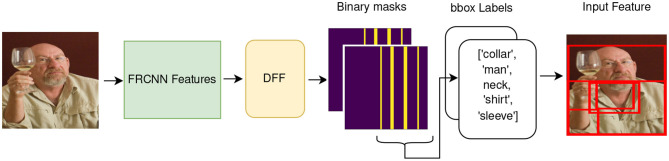
Schematic example of how to generate semantic features with DFF from a FRCNN visual backbone. The index of the highlighted band in the binary mask is used to select the bounding boxes corresponding to objects that compose the input features. However, the bounding boxes highly overlap with each other and cover the majority of the pixels in the image.

FRCNNs are often used as feature extractors in V&L models (Anderson et al., 2018; Tan and Bansal, 2019; Zhang et al., 2021). They extract feature vectors representing bounding boxes of salient objects identified in the image. Similarly to ViT, the FRCNN's activation matrix is a stack of feature vectors, therefore we can extract semantic features, similarly to the method described in Section 4.5.1. However, FRCNNs tend to extract highly overlapping bounding boxes, which results in massively redundant semantic features. This prevents the features from effectively selecting specific semantic content, as they often result in sharing most of the selected area. A schematic example is shown in Figure 13, where although DFF manages to cluster semantically related boxes (like *collar, man, neck, sleeve*), it ends up selecting a large portion of the image in a single input feature.

An excessive amount of overlap among the features affects their capability to identify specific semantic concepts. Thus, we conclude that **DFF can be adapted to FRCNN's features but does not produce the desired results of capturing enough fine-grained semantic concepts to support informative explanations**. In the following subsection, we describe an alternative route toward obtaining semantically meaningful visual regions that can act as features for explaining V&L models, in cases where the visual backbone does not permit an application of bottom-up, unsupervised methods such as DFF.

#### 4.5.3. Beyond DFF: a model-agnostic semantic feature extraction

As shown in the previous sections:

- the full potential of DFF is evident with CNN models;
- it can be adapted to extract features from ViT models, though they are less detailed due to the initial discretization of the image into patches operated by the model;
- it does not produce satisfactory results on FRCNN activations, because of the redundancy of the bounding boxes extracted by the model.

In order to address limitations coming from the visual backbone's architecture (e.g., in the case of FRCNNs), we propose to use STEGO (Hamilton et al., 2022)^2^ a state-of-the-art segmentation model, to extract semantic feature masks. It is unsupervised, meaning that it does not require ground truth labels. As a consequence, the number of features extracted can not be controlled, though in our experiment we observe that it extracts a small number of semantic masks (usually less than 10). This keeps the Shapley value computation low but could limit the number of semantic concepts captured, differently from DFF where the number of features is a controllable hyperparameter.

The biggest advantage of using an off-the-self segmentation model is that it supports the generation of visual explanations, independently of the visual backbone's architecture. On the other hand, we have the downside of no longer relying on the visual backbones' priors, embedded in the captioning model.

**Figure 14:**
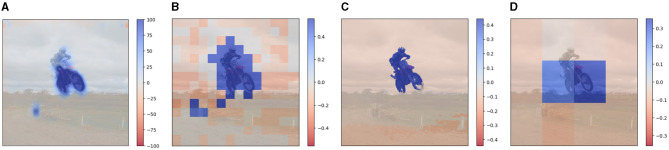
Direct comparison of explanations generated for the caption “riding a dirt bike” from different visual backbones and methods. The two leftmost explanations **(A, B)** are generated from features extracted using DFF and activations of different visual backbones, namely a CNN **(A)** and ViT **(B)**. **(C)** uses semantic masks extracted by a segmentation model (STEGO) and **(D)** uses superpixel features. All the explanations have comparable compute costs, apart from **(C)**, where only 6 features are used. **(A)** DFF (CNN). **(B)** DFF (ViT). **(C)** STEGO. **(D)** Superpixel.

In Figure 14 we directly compare the visual explanations generated by all methods, DFF on CNN and ViT (Figures 14A, B), STEGO (Figure 14C), and superpixel (Figure 14D). All the explanations are generated with similar compute costs, apart from STEGO which uses a smaller amount of features (6). As expected, the explanations generated with STEGO's semantic features are more fine-grained than the others, as the model is trained on the semantic segmentation task. However, they come from an external model and do not necessarily reflect the visual priors of the V&L model itself. Nevertheless, this provides a flexible solution to adapt the explanation of V&L models with visual priors to any visual backbone. Furthermore, any segmentation model can in principle be used.

## 5. Discussion

We have now all the elements needed to answer the question posed at the beginning of this Section. Exploiting the model's visual priors exposes several significant advantages with respect to standard superpixel features. As shown in Section 4.3 input features based on the model's visual priors provide more semantically detailed explanations namely, they succeed in emphasizing salient semantic relevant elements to a higher extent in the image, providing also information regarding the intensity of the area of contribution. The semantic nature of the inputs produces more comprehensive explanations (Section 4.3.1) than standard superpixel features at a lower compute cost, thus being also more efficient (Section 4.3.2).

However, the introduction of semantic visual features introduces several potential issues that we have thoroughly analyzed in this section. From the theoretical point of view, our method requires a relaxation of the feature independence assumption (Section 4.4.2) which however, does not compromise the validity of the underlying core method (i.e., KernelSHAP) as we empirically show that non-disjoint features do not significantly affect the visual explanation. In fact, forcing the disjointness of semantic features leads to misleading visual explanations. Similarly, different sizes in the input feature dimension, do not significantly affect the final contribution, as we show (in Section 4.4.3). Our method is flexible enough to be adapted to Vision Transformers other than CNNs; however, it adapts with difficulty to FasterRCNNs (as discussed in Section 4.5.2). To overcome this issue we propose using an off-the-self semantic segmentation model to extract semantic visual features. In light of our work, which finds its primary motivation is exploiting the model's internal semantic priors, we argue that this solution is not optimal, as it relies on external semantic priors, however, it is a reasonable trade-off that allows us to deal with architectures that do not accommodate DFF to extract such priors.

## 6. Human evaluation

The experiments in the previous section made direct comparisons between our method and superpixel-based explanations for V&L generative models. In this section, we report on an evaluation of human participants aiming to assess the benefits and potential limits of our method for human users.

Evaluating XAI techniques is a notoriously challenging task (e.g., Adebayo et al., 2022; Nauta et al., 2023). Here, we take inspiration from the work of Hoffman et al. (2018) and compare the judgments of participants on three qualities, namely *detail, satisfaction* and *completeness* of explanations generated using the two methods under consideration.

### 6.1. Participants

For the purposes of this study, it is important to source judgments from participants who are knowledgeable about machine learning and explainable AI. Relying on crowd-sourcing is a risky strategy, as there is no guarantee that participants will be in a position to evaluate *explanations* rather than, say, the quality of model outputs. We, therefore, recruited 14 researchers (9 male, 5 female; 9 aged 18–30, 4 aged 31–40, 1 aged 41–50) from our own network. All are researchers in AI-related fields and are familiar with XAI methods. Two of these are senior researchers who obtained their PhD more than 5 years ago; all the others were doctoral students at the time the experiment was run. Six participants are native speakers of English; the remainder are fluent or near-fluent speakers.

### 6.2. Design and materials

We randomly selected 40 images from the HL dataset, for which we generated the corresponding answers to questions. In order to create a more challenging scenario, we framed it into a visual question-answering task, thus for each image, we select one of the available questions and we generate the corresponding caption. Moreover, for each image-caption pair, we generated visual explanations using both DFF and superpixels features.

Each participant was shown the question, the generated answer, the original image, and the visual explanation which can be either generated by DFF or by superpixel. In order to counterbalance the experimental materials, we divided images randomly into two groups, and further assigned participants randomly to two groups. We rotated items through a 2 (participant group) × 2 (image group) Latin square, such that participants in any experimental group evaluated all images, but each image was always seen once and evaluated in only one condition (DFF or superpixel).^3^

The participants were asked to judge explanations based on their agreement to each of the following statements:

- *Detail*: the areas highlighted in the explanation are detailed enough to understand how the model generated the caption;
- *Completeness*: the highlighted areas cover all the regions relevant to the caption;
- *Satisfaction*: based on the areas highlighted in the explanation I feel that I understand how the system explained makes its decisions.

Responses to each dimension were given on a Likert scale from 1 to 5, where 1 corresponds to the total agreement and 5 to total disagreement. For the full evaluation form see the Supplementary material.

## 7. Results

**Figure 15:**
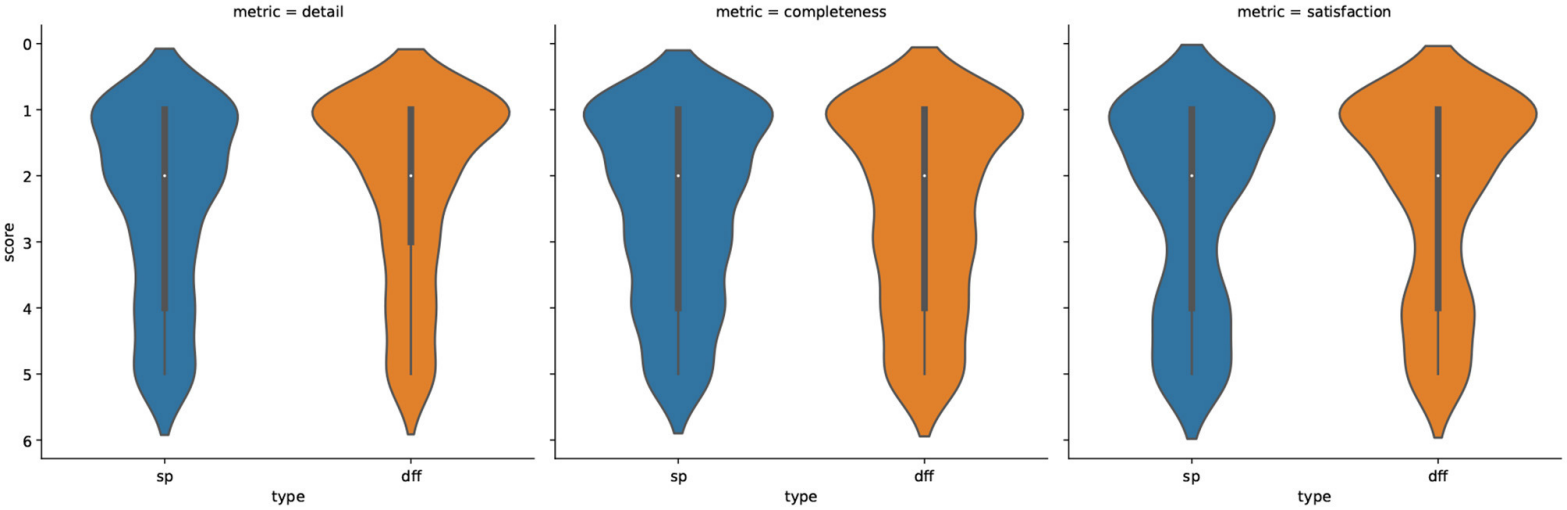
Distribution of the Likert scores obtained in the human evaluation for *detail, completeness* and *satisfaction* for both DFF in (orange) and superpixel (in blue). The lower the score the higher the rating.

As shown in Figure 15, DFF-based explanations (in orange) are considered on par with superpixel-based explanations (in blue) in terms of completeness, but at the same time, they are considered more detailed and more satisfactory for human judges. Thus, the score distributions for detail and satisfaction are more skewed toward lower (better) scores for the detail and satisfaction criteria. More detailed statistics are reported in the Supplementary material.

Although the superpixel and DFF methods differ in the judged level of detail of the explanations, they yield attributions that are similarly located in the input image. This is in part due to the fact that in both cases, we are using the same feature attribution method, namely Kernel SHAP. However, in some cases, we observe a certain degree of divergence in the visual explanation, meaning that the two methods assign opposite attributions to similar regions. An example is reported in the Supplementary material.

This is probably due to the particular configuration of features selected by both methods, which in some instances might select insufficiently detailed regions, preventing the method from highlighting the semantically relevant areas of the image.

In order to quantify this phenomenon we manually inspected the 40 samples used in the human evaluation. We found that around 10% of the explanations diverged to some extent between the two feature selection methods. We analyzed separately this sub-sample of divergent explanations. We find that the average scores given by experimental participants for this subset are overall slightly worse (higher) than the full results (see Supplementary material for details). Nevertheless, the trends observed in relation to Figure 15 for the three evaluation criteria still hold. This suggests that this phenomenon does not significantly affect the participants' judgments, except for a slight drop in the perceived quality of the explanations.

Moreover, in qualitative feedback given by participants, some declared that in some instances, their assessment was affected by the correctness of the caption, which in some cases was considered wrong or partially inaccurate. We quantified the inaccuracy of the caption by computing their lexical and semantic similarity with respect to the reference captions, using respectively, BLEU (Papineni et al., 2002) and Sentence-Bert (Reimers and Gurevych, 2019). We computed the Pearson correlation (Cohen et al., 2009) between the Likert scores and the lexical and semantic similarity previously computed. We find that the Likert scores slightly but not significantly positively correlate with both lexical and semantic similarity (ρ = −0.023 for lexical similarity and ρ = −0.004 for semantic similarity).^4^ This suggests that despite the fact that participants did note the quality of the captions, this did not significantly affect their judgments of the explanations.

In conclusion, we found that assessing visual explanations is a hard task even for specialists in the field. We observed a relatively low inter-annotator agreement for both groups in the Likert judgments [Krippendorff's α = 0.23 (Krippendorff, 2004)]. However, besides possible confounding factors, like inaccuracies of the captions and divergent explanations, the DFF-based explanations are generally perceived as higher quality explanations than superpixel-based ones.

## 8. Limitations

Some potential limitations of our method arise from the adaptations we have made to Kernel SHAP as the core explainability method.

First, we observe that semantic features extracted with DFF may overlap with each other and therefore may generate non-disjoint features. This directly affects the assumption of feature independence, which is a theoretical requirement for SHAP. However, we study (in Section 4.4.2 the extent to which this phenomenon is present and how it affects the outcome of our method finding that it does not significantly affect our visual explanations, thus, our method features a relaxation of this assumption.

Second, semantic feature extraction, namely DFF, is designed to extract visual priors from CNN-based models. In Section 4.5.3, we show that this method can successfully adapt to Vision Transformers, but not to FasterRCNNs. To overcome this limitation we propose to use an off-the-shelf segmentation method to extract semantic features. This solution supports visual explanation, independently of the visual backbone's architecture. However, in view of the motivation of our work, whose main goal is to exploit the model's visual priors to explain its own predictions, we argue that this solution is not optimal, as it relies on external visual priors (i.e., a third-party semantic segmentation model),

Ultimately, as shown in Section 6, we validate our method through a human evaluation. We leverage experts in AI and we design our evaluation in an unambiguous way for our annotators. However, we are aware that evaluating visual explanations for humans can be a hard task. In particular, the task of evaluating XAI is ambiguous, since evaluators are asked to judge the quality of explanations, which is in principle distinct from the quality of model outputs (that is, one can have a satisfactory explanation of an incorrect or infelicitous output). As the qualitative feedback from our evaluation suggests, keeping output quality and explanation quality separate is not always an easy task and this may influence the evaluation outcomes.

## 9. Conclusions

In this work, we proposed an explainability framework to bridge the gap between multimodality and explainability in image-to-text generative tasks exploiting textual and visual semantics.

Our method is developed around SHAP, as it provides a model-agnostic solution with solid theory and desirable properties. We design our approach to address certain crucial limitations of current approaches.

First, SHAP-based methods are rarely employed to explain large models as they are extremely expensive to compute. Our solution is efficient and allows an accurate approximation of the Shapley values.

Second, we overcome the limitations of current token-by-token explanations by proposing sentence-based explanations exploiting semantic textual variations which are also more efficient to compute.

Finally, based on the rationale that a model's generative outputs should be explained with reference to the knowledge encoded by the visual backbone, we propose an unsupervised method to extract semantically informative visual features. Using these features rather than superpixels means that we obtain explanations which are cheaper (insofar as more can be gleaned from fewer features) but also more intuitive, especially when compared to superpixel-based approaches.

We show that our method can be employed with different visual-backbones architectures like CNN and Vision Transformers. In the case of visual backbones for which desirable results cannot be produced, such as FasterRCNN-based models, we propose an alternative solution based on a semantic segmentation model, to generate semantically meaningful input features.

Through a human evaluation, we show that using semantic priors improves the perceived quality of the explanation, resulting in more detailed and satisfactory explanations than superpixels though matching the same level of completeness.

Moreover, our framework is totally modular and it can co-exist with a wide range of possible configurations for all of its components. For example, it is possible to produce token-by-token explanations and still rely on DFF to extract visual features. The core method, Kernel SHAP, can be replaced with another SHAP-based method, and the visual features can be extracted with one of the proposed methods or with any other method of choice.

## Data availability statement

Publicly available datasets were analyzed in this study. This data can be found here: https://github.com/michelecafagna26/HL-dataset.

## Ethics statement

The studies involving humans were approved by University of Malta Research Ethics Committee. The studies were conducted in accordance with the local legislation and institutional requirements. The participants provided their written informed consent to participate in this study.

## Author contributions

MC implemented the method and run the experiments and the evaluation. AG and LR-B supervised the experiments and the method design. MC and AG designed the evaluation and KD and LR-B gave feedback with a special eye to evaluation. MC and AG contributed to the paper writing. All authors contributed to the article and approved the submitted version.

## Footnotes

^1^ https://github.com/OFA-Sys/OFA

^2^At the time of this work, STEGO was the state-of-the-art model for semantic segmentation. However, the approach proposed here is agnostic as to the segmentation model used. For example, Segment Anything (Kirillov et al., 2023), a more recent model proposed after the present experiments were completed could yield better results.

^3^In the end, the experiment was completed by 8 participants in one group, and 6 in the other.

^4^Note that since in the Likert score, 1 is the maximum agreement and 5 the minimum, a positive correlation corresponds to a negative ρ.
