# Supplementary material for: Interpreting vision and language generative models with semantic visual priors
Source: Front Artif Intell. 2023 Sep 25;6:1220476. doi: 10.3389/frai.2023.1220476 (PMC10561255; doi:10.3389/frai.2023.1220476)
Supplement: Supplementary file 1 [file Data_Sheet_1.PDF]

Supplementary Material

1 DATA EXAMPLE

Below is an example image, with corresponding captions, from the HL Dataset used in the experiments.

Image	Axis	Caption
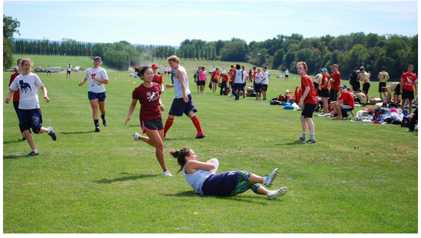	scene	at a sport field
	action	they are playing a sport
	rationale	they are having fun
	object-centric (COCO)	A woman has fallen on the ground in a field.

Table S1. Example of High-Level captions. For each of the dimensions of *scene, action, rationale*, we show one of the three captions available in the dataset, combined with the object-centric captions from COCO.

2 HUMAN EVALUATION RESULTS

This section reports the details of the human evaluation discussed in the main paper. The instructions given to participants are shown in Figure S1. Figure S2 is an example of the form used by participants to evaluate each item. Table S2 gives full descriptive statistics for the results reported in the main paper. Figure S3 shows an example from the subset of cases where the superpixel and the DFF-based methods highlight different regions of the image as contributing to the model output.

Type	Metric	Mean	Std	Median
SP	completeness	2.48	1.38	2.0
	detail	2.46	1.42	2.0
	satisfaction	2.51	1.51	2.0
DFF	completeness	2.50	1.45	2.0
	detail	2.18	1.41	2.0
	satisfaction	2.32	1.48	2.0

Table S2. Results of the human evaluation, for superpixel-based (SP) and DFF-based (DFF) visual explanation. We report the mean, the standard deviation (std), and the median of the Likert scores. The lower the score the more positive the rating.

Below we will introduce you to some concepts to help you in the evaluation process

1) Our **image captioning** system works by generating **answers to questions**. These answers can be full sentences or even simple words or phrases. Item 1

You can see an example below:

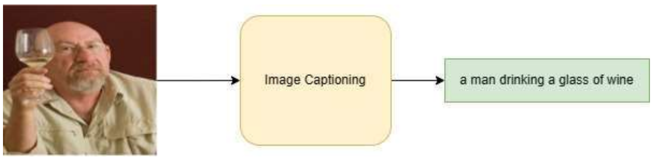

2) A **visual explanation highlights** the **areas** of the image which **positively or negatively contribute** to generate the **caption**. This is helpful to understand how the system uses the information depicted in the image.

Below you can see the **visual explanation** of the caption ***"a man drinking a glass of wine"*** *, generated by a* captioning system**.**

Overall,
the **blue** areas **positively contribute** to produce the caption;
the **red** areas **negatively contribute** to produce the caption.

Always refer to the color bar on the right-hand side of the explanation as it gives you a numerical reference to understand the scale of intensity of the color, which might change from an explanation to another.

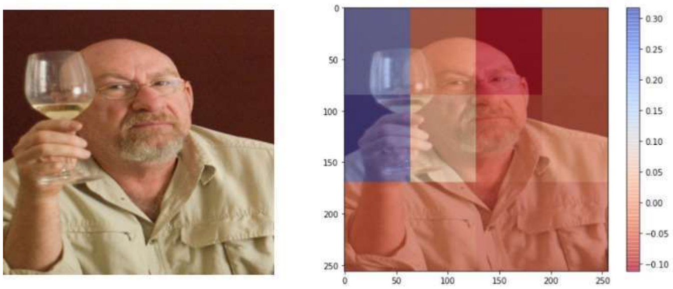

3) What are we evaluating?
We are going to evaluate the following properties by asking to what extent you agree with these statements

- **Detail**: the areas highlighted in the explanation are detailed enough to understand how the model generated the caption
- **Completeness**: the highlighted areas cover all the regions relevant for the caption
- **Satisfaction**: based on the areas highlighted in the explanation I feel that I understand how the system explained makes its decisions

Figure S1. Instruction presented to the participants of the human evaluation.

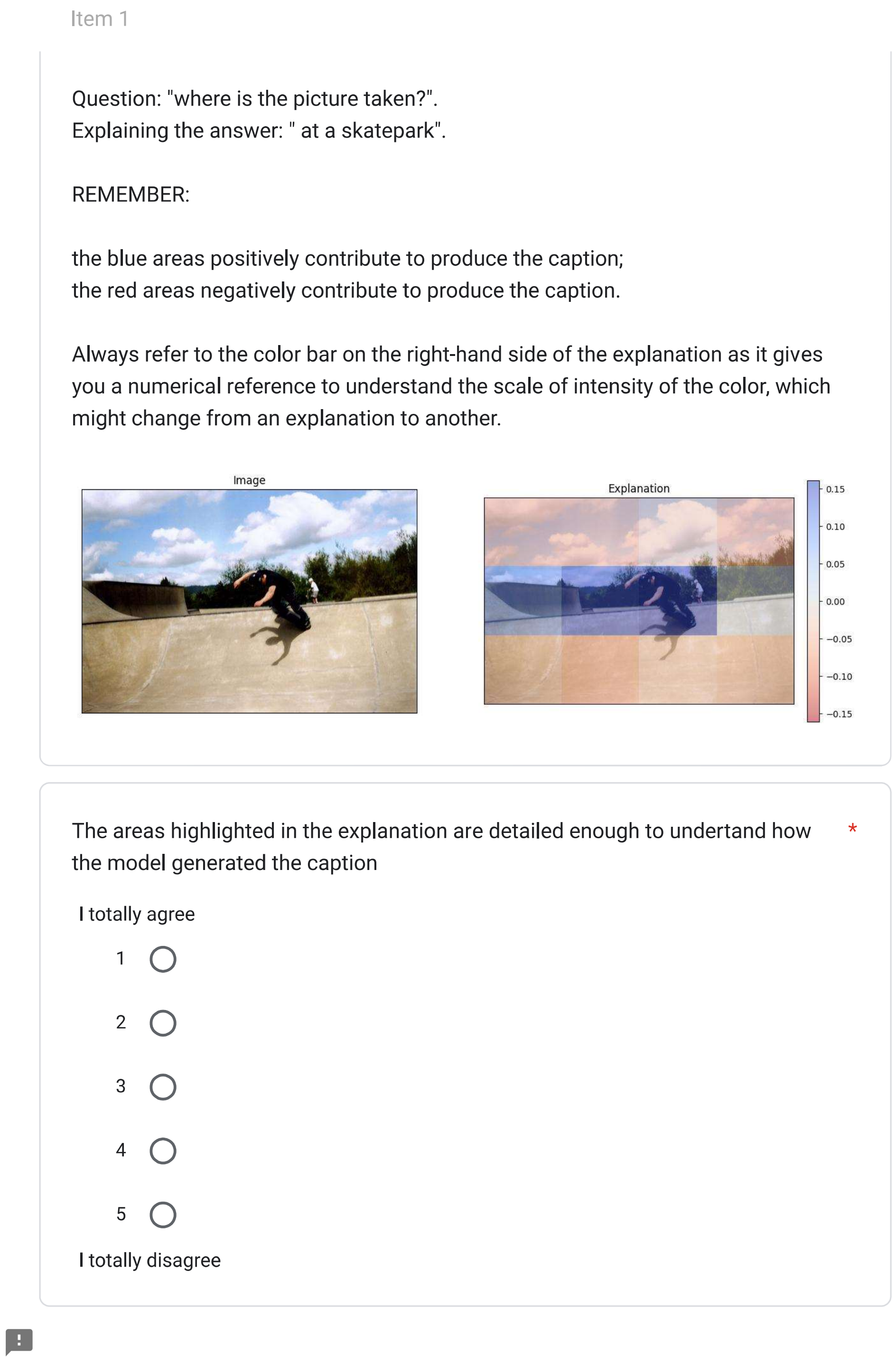
Item 1

Question: "where is the picture taken?".
Explaining the answer: " at a skatepark".

REMEMBER:

the blue areas positively contribute to produce the caption;
the red areas negatively contribute to produce the caption.

Always refer to the color bar on the right-hand side of the explanation as it gives you a numerical reference to understand the scale of intensity of the color, which might change from an explanation to another.

The areas highlighted in the explanation are detailed enough to undertand how the model generated the caption *

I totally agree

1
2
3
4
5

I totally disagree

Figure S2. Example of an item presented to the participants of the human evaluation. It shows the question, the generated caption, the original image, and the visual explanation. The participant is asked to measure the agreement with three statements related to *detail, completeness* and *satisfaction*. In this Figure, we show the statement related to *detail*.

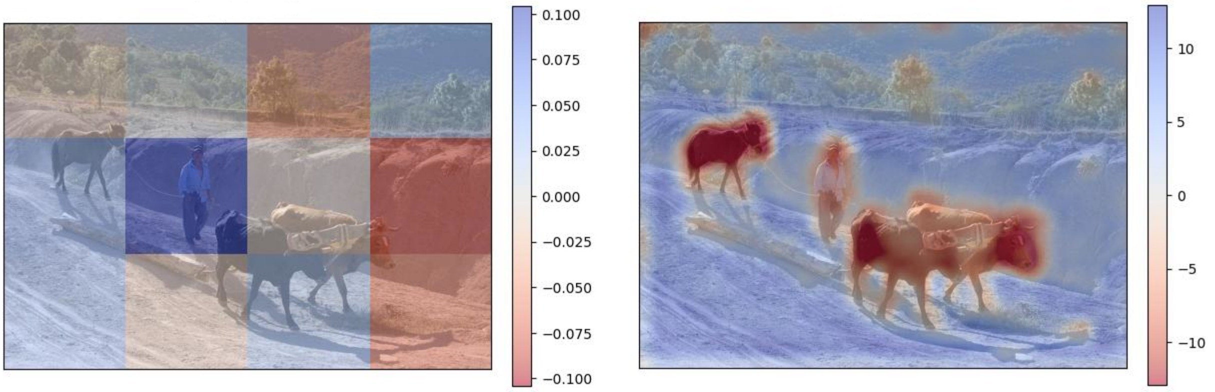

Figure S3. Comparison of divergent explanations for the question: ”Where is the picture taken?” and generated caption: ”on a dirty road”, obtained from superpixel features (on the left) and DFF features (on the right).
